# Prognostic impact of dose reduction in androgen receptor pathway inhibitors for castration-resistant prostate cancer

**DOI:** 10.1016/j.prnil.2021.10.001

**Published:** 2021-10-30

**Authors:** Shigetomo Yamada, Masaki Shiota, Leandro Blas, Takashi Matsumoto, Eiji Kashiwagi, Ario Takeuchi, Junichi Inokuchi, Ken-ichiro Shiga, Akira Yokomizo, Masatoshi Eto

**Affiliations:** a Department of Urology, Graduate School of Medical Sciences, Kyushu University, Fukuoka, Japan; b Department of Urology, Harasanshin Hospital, Fukuoka, Japan

**Keywords:** Abiraterone, Androgen receptor pathway inhibitor, Castration-resistant prostate cancer, Dose reduction, Enzalutamide

## Abstract

**Background:**

Androgen receptor pathway inhibitors (ARPIs) such as abiraterone and enzalutamide have been shown to prolong survival in patients with advanced prostate cancer. However, there is limited evidence on the anticancer effect of a reduced dose of ARPIs. This study compared the prognosis in patients with chemotherapy-naïve castration-resistant prostate cancer (CRPC) between ARPI treatment with standard dose and treatment with reduced dose.

**Methods:**

Japanese patients who were treated with ARPI as first-line treatment for CRPC between 2014 and 2018 were included. The associations between dose reduction and clinicopathological factors, progression-free survival, and overall survival were investigated.

**Results:**

Of the 162 patients included, 33 (20.4%) patients had their dose reduced during ARPI treatment. In the multivariate analysis, higher PSA, abiraterone treatment, and dose reduction were significant prognostic factors for progression-free survival (PFS); however, dose reduction was not associated with overall survival. In the enzalutamide-treated group, the median PFS was 12.1 months (95% CI, 8.5–21.4 months) in the standard-dose group and 7.2 months (95% CI, 5.0–11.5 months) in the reduced-dose group (*P* = 0.038).

**Conclusion:**

This study suggests inferior oncological outcome when treated with reduced-dose ARPI for CRPC. Full-dose administration of ARPI for CRPC may be appropriate if feasible.

## 1 Introduction

Androgen deprivation therapy (ADT) has been the standard treatment for advanced prostate cancer since 1941.^1^ However, most advanced prostate cancers eventually relapse as castration-resistant prostate cancer (CRPC). Androgen receptor pathway inhibitors (ARPIs), such as abiraterone acetate and enzalutamide, have been shown to prolong freedom from progression and survival when used for CRPC in post-chemotherapy and chemotherapy-naïve settings.^2^, ^3^, ^4^, ^5^ In phase I studies on enzalutamide, the PSA decrease was dose-dependent from 30 to 150 mg.^6^^,^^7^ Accordingly, the standard dose of enzalutamide was determined as 160 mg daily in subsequent clinical trials. Similarly, in phase I studies on abiraterone, from 250 to 2,000 mg doses in fasted and fed men were examined, and then, the standard dose of abiraterone for further development was determined as 1,000 mg in the fasted state.^8^^,^^9^

ARPIs increase the risk of cardiac, metabolic, and musculoskeletal disorders.^10^^,^^11^ Particularly, abiraterone induces hepatobiliary disorders while enzalutamide induces psychiatric disorders.^10^ These adverse effects (AEs) may lead to a reduction of the administered dose. Usually, dose reduction can result in a detriment of anticancer effects in several anticancer treatment regimens.^12^ However, so far, there is limited evidence on the anticancer effect of a reduced dose of ARPIs. Therefore, we compared the prognosis of patients with chemotherapy-naïve CRPC using ARPIs in standard dose versus reduced dose.

## 2 Patients and Methods

### 2.1 Patients

This study retrospectively included Japanese men who received ARPI (abiraterone acetate or enzalutamide) as first-line treatment, life-prolonging agent, for CRPC at Kyushu University Hospital (Fukuoka, Japan) and Harasanshin Hospital (Fukuoka, Japan) from May 2014 to December 2018.^13^^,^^14^ The study was approved by the review board of each institution. Patients without histopathological diagnosis of adenocarcinoma of the prostate were excluded. Clinical stage was determined according to the uniform TNM criteria, based on the results of digital rectal examination, transrectal ultrasonography, magnetic resonance imaging, computed tomography, and bone scintigraphy.^15^ All patients had been treated with ADT before progressing to CRPC. CRPC was diagnosed based on increased prostate-specific antigen (PSA) levels and/or radiographic progression by the physician's judgment.

### 2.2 Treatment

As a standard-dose treatment, either abiraterone (1,000 mg/day) in fasted state with prednisolone (10 mg/day), or enzalutamide (160 mg/day) was administered as reported previously.^2^, ^3^, ^4^, ^5^ Both abiraterone and enzalutamide were approved for CRPC with or without metastasis in Japan. The choice of enzalutamide or abiraterone was determined by the physician's discretion. Castration status was maintained by surgical or continuous medical castration with a luteinizing hormone-releasing hormone antagonist (degarelix acetate) or agonist (leuprorelin acetate or goserelin acetate), during treatment with ARPI. Treatment with ARPI was discontinued at the physician's discretion based on disease progression, AEs, or patient's refusal. Dose reduction was determined by the physician when treatment was initiated or when AEs were detected.

### 2.3 Endpoints

Progression-free survival (PFS) and overall survival (OS) were defined from the date of initiation of ARPI for CRPC to the date of the event. Disease progression was determined by PSA increase of >2 ng/mL and 50% increase over the nadir, or radiographic progression by the emergence of two new lesions or progression of one or more known lesions, based on the Response Evaluation Criteria in Solid Tumors (RECIST).^16^ Disease progression and death due to any cause were defined as the end event for PFS and OS, respectively.

### 2.4 Statistical analysis

All statistical analyses were performed using JMP14 software (SAS Institute, Cary, NC, USA). Categorical and continuous data were compared by Pearson's chi-square and Wilcoxon's rank-sum tests, respectively. Survival analysis was performed using the Kaplan–Meier method and compared between groups using the log-rank test. Cox proportional hazards model was used to estimate the hazard ratio (HR). All tests were two-sided, and *P* < 0.05 was considered statistically significant.

## 3 Results

Clinicopathological characteristics of the 162 patients are shown in Table 1. The median age was 75 years (interquartile range [IQR], 70–82 years), and the median PSA at pre-treatment was 12.1 ng/mL (IQR, 5.1–41.9 ng/mL). The median time to CRPC was 18.0 months (IQR, 9.6–35.8 months). Most patients had a Gleason score >8 and presented bone metastases. As first-line treatment for CRPC, 57 patients received abiraterone, and 105 men received enzalutamide. Among them, 33 (20.4%) patients had their dose reduced during ARPI treatment. Treatment was initiated in 24 patients with reduced dose whereas the administration dose was reduced in nine patients due to AEs. Older age, higher PSA level, and enzalutamide treatment were associated with dose reduction (Table 1).

**Table 1:** Patients' characteristics

	All (*n* = 162)	Dose reduction		
		Absence (*n* = 129)	Presence (*n* = 33)	*P*-value
Median age, years (IQR)	75 (70–82)	74 (69–81)	82 (74–86)	0.0009^a^
Median PSA, ng/ml (IQR)	12.1 (5.1–41.9)	10.2 (4.4–37.7)	23.1 (11.9–47.4)	0.010^a^
Median time to CRPC, months (IQR)	18.0 (9.6–35.8)	17.0 (9.8–28.7)	20.4 (8.1–81.0)	
Gleason score, *n* (%)				
≤8	61 (38.4%)	50 (39.4%)	11 (34.4%)	
>8	98 (61.6%)	77 (60.6%)	21 (65.6%)	0.60
Not available	3	2	1	
Prior local therapy, *n* (%)				
Absence	103 (63.6%)	80 (62.0%)	23 (69.7%)	
Radical prostatectomy	22 (13.6%)	19 (14.7%)	3 (9.1%)	
Radiation	37 (22.8%)	30 (23.3%)	7 (21.2%)	0.64
Bone metastasis, *n* (%)				
Absence	56 (34.6%)	43 (33.3%)	13 (39.4%)	
Presence	106 (65.4%)	86 (66.7%)	20 (60.6%)	0.51
Visceral metastasis, *n* (%)				
Absence	151 (93.2%)	122 (94.6%)	29 (87.9%)	
Presence	11 (6.8%)	7 (5.4%)	4 (12.1%)	0.17
Androgen receptor pathway inhibitor, *n* (%)				
Abiraterone	57 (35.2%)	52 (40.3%)	5 (15.2%)	
Enzalutamide	105 (64.8%)	77 (59.7%)	28 (84.8%)	0.0069^a^

IQR, interquartile range; PSA, prostate-specific antigen; CRPC, castration-resistant prostate cancer.

a Statistically significant.

**Fig. 1:**
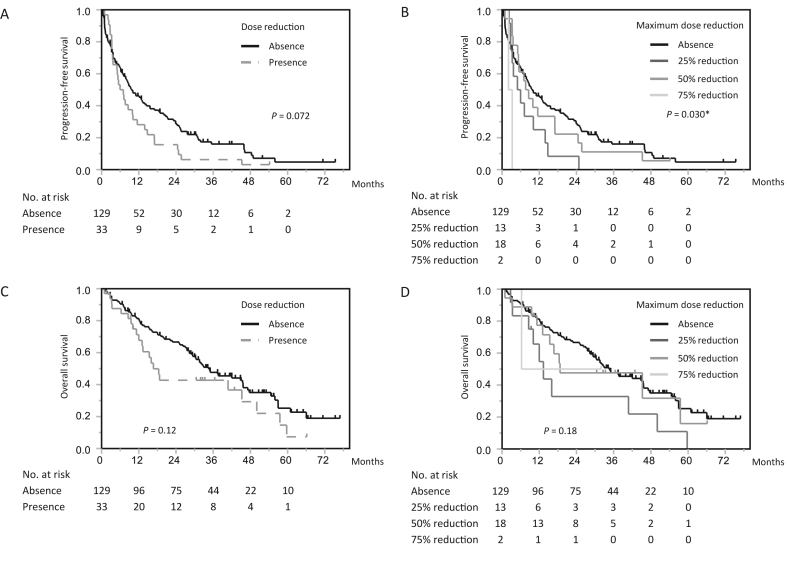
Progression-free survival (PFS) and overall survival (OS) in patients stratified by the dose of androgen receptor pathway inhibitor administration. (A) and (B) Kaplan–Meier survival curves of PFS when stratified by dose reduction (A) and maximum dose-reduction rate (B). (C) and (D) Kaplan–Meier survival curves of OS when stratified by dose reduction (C) and maximum dose-reduction rate (D).

The median follow-up time for men alive at censoring date was 35.0 months (IQR, 18.8–49.7 months). During follow-up, 133 patients (82.1%) experienced disease progression, and 97 patients (59.9%) died from any cause. The median PFS and OS were 8.7 months (95% CI, 6.7–11.5 months) and 32.9 months (95% CI, 27.5–44.8 months), respectively. When patients were divided into two groups according to the dose reduction, the median PFS was 9.6 months (95% CI, 7.0–14.0 months) in the standard-dose group and 6.5 months (95% CI, 3.5–10.1 months) in the reduced-dose group (*P* = 0.072, Fig. 1A). When the reduced-dose group was subdivided by maximum dose-reduction rate, the median PFS was 5.5 months (95% CI, 2.7–13.9 months) in 25% reduced-dose group, 8.1 months (95% CI, 5.0–17.0 months) in 50% reduced-dose group, and 2.7 months (95% CI, 2.1–3.3 months) in 75% reduced-dose group (*P* = 0.030, Fig. 1B). The median OS was 34.5 months (95% CI, 29.1–45.6 months) in standard-dose group and 16.7 months (95% CI, 12.0–45.2 months) in the reduced-dose group (*P* = 0.12, Fig. 1C). When subdivided by maximum dose-reduction rate, the median OS was 13.3 months (95% CI, 3.3–50.1 months) in 25% reduced-dose group, 18.7 months (95% CI, 13.1–57.5 months) in 50% reduced-dose group, and 6.3 months (95% CI, 6.3 months – not reached) in 75% reduced-dose group (*P* = 0.18, Fig. 1D). In univariate analysis, higher PSA and abiraterone treatment, but not dose reduction was significantly associated with shorter PFS (Table 2). In the multivariate analysis, higher PSA, abiraterone treatment, and dose reduction were significant prognostic factors for PFS (Table 2). In univariate and multivariate analyses, dose reduction was not associated with OS (Table 3).

**Table 2:** Associations between clinicopathological parameters and progression-free survival

Variable	Univariate analysis			Multivariate analysis		
	HR	95% CI	*P*-value	HR	95% CI	*P*-value
Pretreatment age (per 10 years)	1.20	0.97–1.50	0.10	1.25	0.97–1.62	0.09
Pretreatment PSA (per 100 ng/ml)	1.02	0.998–1.03	0.020^a^	1.02	0.996–1.03	0.036^a^
Time to CRPC (per 12 months)	0.97	0.91–1.03	0.30	0.97	0.90–1.03	0.34
Gleason score						
≤8	ref	-	-	ref	-	-
>8	0.96	0.68–1.38	0.84	0.92	0.64–1.33	0.66
Prior local therapy						
Absence	ref	-	-	ref	-	-
Radical prostatectomy	0.65	0.38–1.11	0.12	0.90	0.51–1.61	0.72
Radiation	1.09	0.72–1.64	0.69	1.46	0.90–2.36	0.13
Bone metastasis						
Absence	ref	-	-	ref	-	-
Presence	1.30	0.90–1.86	0.16	1.22	0.79–1.88	0.36
Visceral metastasis						
Absence	ref	-	-	ref	-	-
Presence	1.33	0.62–2.86	0.46	1.82	0.82–4.01	0.14
Androgen receptor pathway inhibitor						
Abiraterone	ref	-	-	ref	-	-
Enzalutamide	0.68	0.47–0.97	0.034^a^	0.61	0.41–0.91	0.015^a^
Dose reduction						
Absence	ref	-	-	ref	-	-
Presence	1.45	0.96–2.17	0.075	1.64	1.03–2.59	0.036^a^

HR, hazard ratio; CI, confidence interval; PSA, prostate-specific antigen; CRPC, castration-resistant prostate cancer.

a Statistically significant.

**Table 3:** Associations between clinicopathological parameters and overall survival

Variable	Univariate analysis			Multivariate analysis		
	HR	95% CI	*P*-value	HR	95% CI	*P*-value
Pretreatment age (per 10 years)	1.59	1.20–2.12	0.0013^a^	1.68	1.21–2.31	0.0017^a^
Pretreatment PSA (per 100 ng/ml)	1.03	0.99–1.05	0.035^a^	1.03	0.998–1.05	0.071
Time to CRPC (per 12 months)	0.94	0.86–1.01	0.12	0.93	0.85–1.02	0.12
Gleason score						
≤8	ref	-	-	ref	-	-
>8	0.98	0.65–1.48	0.93	0.98	0.65–1.49	0.93
Prior local therapy						
Absence	ref	-	-	ref	-	-
Radical prostatectomy	0.47	0.24–0.92	0.027^a^	0.76	0.37–1.55	0.45
Radiation	0.86	0.52–1.40	0.54	1.07	0.62–1.86	0.81
Bone metastasis						
Absence	ref	-	-	ref	-	-
Presence	1.36	0.88–2.10	0.17	1.03	0.63–1.70	0.90
Visceral metastasis						
Absence	ref	-	-	ref	-	-
Presence	1.22	0.53–2.79	0.64	2.10	0.88–5.03	0.095
Androgen receptor pathway inhibitor						
Abiraterone	ref	-	-	ref	-	-
Enzalutamide	0.65	0.43–0.98	0.038^a^	0.58	0.37–0.91	0.017^a^
Dose reduction						
Absence	ref	-	-	ref	-	-
Presence	1.46	0.91–2.36	0.12	1.52	0.90–2.59	0.12

HR, hazard ratio; CI, confidence interval; PSA, prostate-specific antigen; CRPC, castration-resistant prostate cancer.

a Statistically significant.

**Fig. 2:**
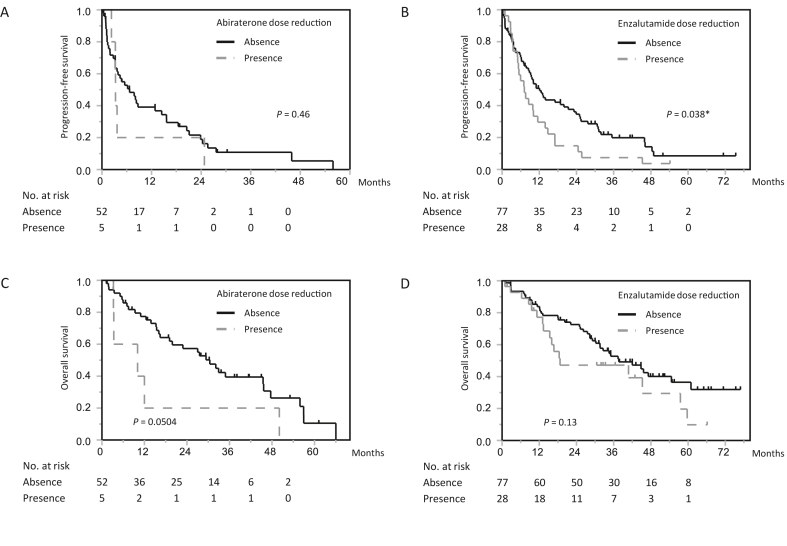
Progression-free survival (PFS) and overall survival (OS) in patients stratified by the dose of abiraterone or enzalutamide administration. (A) and (B) Kaplan–Meier survival curves of PFS when stratified by dose reduction of abiraterone (A) and enzalutamide (B). (C) and (D) Kaplan–Meier survival curves of OS when stratified by dose reduction of abiraterone (C) and enzalutamide (D).

The prognostic impact of dose reduction of the therapeutic agent used as first-line treatment for CRPC was assessed. When patients were treated with abiraterone, the median PFS was 6.7 months (95% CI, 3.2–14.5 months) in the standard-dose group and 3.3 months (95% CI, 2.3–24.8 months) in the reduced-dose group (*P* = 0.46, Fig. 2A). When patients were treated with enzalutamide, the median PFS was 12.1 months (95% CI, 8.5–21.4 months) in the standard-dose group and 7.2 months (95% CI, 5.0–11.5 months) in the reduced-dose group (*P* = 0.038, Fig. 2B). When using abiraterone, the median OS was 30.5 months (95% CI, 16.4–45.6 months) in the standard-dose group and 10.1 months (95% CI, 3.2–50.1 months) in the reduced-dose group (*P* = 0.0504, Fig. 2C). When using enzalutamide, the median OS was 37.6 months (95% CI, 30.2–54.6 months) in the standard-dose group and 18.7 months (95% CI, 13.3–57.5 months) in the reduced-dose group (*P* = 0.13, Fig. 2D).

## 4 Discussion

This study showed that dose reduction of ARPI, particularly enzalutamide, was associated with shorter PFS compared with the patients treated with the standard dose of ARPI. In addition, a similar trend on the association with OS was obtained, although statistical significance was not reached probably due to the small number of cases. Similarly, consistent trends of dose reduction on PFS and OS were observed in both abiraterone and enzalutamide. Taken together, these findings suggest that dose reduction of ARPI, mainly enzalutamide, may lead to a reduction in its anticancer effect, resulting in a poor oncological outcome. Consistently, Freedland et al reported that dose reduction was associated with a significantly higher risk of PSA progression when administering abiraterone or enzalutamide in a group of 6,069 veterans with metastatic CRPC.^17^ Meanwhile, Vinh-Hung et al reported that the PSA decline and PFS were comparable between low-dose (≤80 mg/day) and standard-dose enzalutamide among patients ≥75 years old.^18^ However, this study retrospectively included only 59 elderly patients, of whom 16 received low-dose and 43 standard-dose therapies, suggesting insufficient statistical power.^18^ Also, Petrioli et al reported that low-dose abiraterone (750 mg/day) in the fasted state among patients ≥85 years old was modestly effective and well tolerated.^19^ However, this single-arm study included only 26 patients, and the comparison with standard-dose treatment was not performed.

Interestingly, abiraterone absorption is improved when taken with low-fat and high-fat meals.^20^ Similar pharmacokinetics were reported for 250–500 mg of abiraterone with high-fat meals and 1,000 mg in the fasted state.^15^ Then, costs may also be decreased by reducing doses when the agent is administered with a meal. Accordingly, a prospective randomized phase II study comparing standard dose (1,000 mg/day) at fasted state and low dose (250 mg/day) with a low-fat meal showed comparable efficacy.^20^ Afterward, the National Comprehensive Cancer Network included low-dose abiraterone (250 mg/day) with a meal as an alternative treatment to full-dose abiraterone (1,000 mg/day) in the fasted state, especially when resources were limited.^21^ However, these findings on the study of low-dose abiraterone with a meal indicate that maintaining dose intensity is important for achieving the expected anticancer effect. This study did not show statistical significance when only patients treated with abiraterone were analyzed, which may a result of insufficient statistical power. Otherwise, the effects of a meal on abiraterone absorption and genetic polymorphisms in *HSD3B1* and *SRD5A2* might also impact on the metabolism of abiraterone and its anticancer properties.^22^^,^^23^

Furthermore, this study showed that abiraterone as first-line treatment for CRPC, in addition to pretreatment PSA level, was associated with inferior freedom from progression and survival. However, previous prospective phase 2 studies showed comparable prognosis including PFS in first-line treatment and OS when those agents were used sequentially.^24^ Then, the inferior prognosis, when patients received abiraterone as first-line treatment for CRPC, seems to be due to a bias such as frequent use of enzalutamide for non-metastatic CRPC based on level 1 evidence.^25^

The main limitations of this study are its retrospective design and its sample size. In addition, some data are missing data; information on concomitant medications and supplements was not available, which may affect serum and tissue levels of abiraterone and enzalutamide.^26^, ^27^, ^28^, ^29^ Another important point was that treatment for CRPC (abiraterone or enzalutamide) was decided by each physician, and subsequent treatments were not defined. We cannot exclude the possibility that the unfavorable outcome with a reduced-dose treatment derived from biases from missing information such as poor performance status and comorbidity status. Therefore, the findings obtained in this study need to be explored in other studies in the future.

In conclusion, this study suggests that there may be an inferior oncological outcome when patients with CRPC are treated with reduced-dose ARPI. Therefore, full-dose administration of ARPI for CRPC may be appropriate, if physiologically and economically feasible.

## Grant support

None.

## Conflicts of interest

Masaki Shiota, Akira Yokomizo, and Masatoshi Eto have received honoraria from Janssen Pharma, Astellas Pharma, and Sanofi.
